# Erectile dysfunction predictors in hypertensives at a primary care clinic in Southern Nigeria

**DOI:** 10.4102/phcfm.v14i1.3244

**Published:** 2022-06-30

**Authors:** Oluwagbenga Ogunfowokan, Sylvia I. Ezemenahi, Anthonia N. Alabi, Adesuwa Q. Aigbokhaode, Bamidele A. Ogunfowokan

**Affiliations:** 1 Faculty of Medicine, Lincoln University, Petaling Jaya Selangor, Malaysia; 2 Department of Family Medicine, Faculty of Medicine, Nnamdi Azikiwe University Teaching Hospital, Nnewi, Nigeria; 3 Department of Family Medicine, Faculty of Medicine, Nnamdi Azikiwe University, Awka, Nigeria; 4 Department of Family Medicine, University of Ilorin, Ilorin, Nigeria; 5 Department of Community Medicine, Federal Medical Centre, Asaba, Nigeria; 6 Department of Family Medicine, The Ark Medical Centre, Asaba, Nigeria

**Keywords:** erectile dysfunction, hypertension, men, education level, diuretics, sexual dysfunction, International Index of Sexual Health Inventory

## Abstract

**Background:**

Erectile dysfunction (ED) has been described as an important public health problem by the National Institutes of Health Consensus Development Conference Panel. It causes significant distress in men and dysfunctional family dynamics.

**Aim:**

This study sought to identify the relationship between level of education and ED amongst hypertensive men (aged 30–89 years) attending outpatient clinics (OPCs) at the Federal Medical Centre (FMC), Asaba.

**Setting:**

This study was conducted in the OPCs at FMC, Asaba, Delta State, Nigeria.

**Methods:**

After obtaining approval from the ethics and research committees in Asaba, 184 consenting hypertensive men who met the eligibility criteria were selected by systematic random sampling to participate in the study from October 2015 to January 2016. This study was a cross-sectional survey. Data were collected with a semistructured, interviewer-administered questionnaire adopted from the International Index of Sexual Health Inventory for Men. The study complied with the principles of Helsinki and Good Clinical Practice.

**Results:**

The mean age ± standard deviation and range of the respondents were 55.1 (±12.4) and 30–89 years, respectively. On logistic regression, higher level of education (secondary school and above) (odds ratio [OR] = 15.943, 95% confidence interval [CI] = 1.517–167.502) was found to be a predictor of ED amongst the study participants.

**Conclusion:**

This study showed that formal education up to secondary level and use of diuretics were significantly associated with ED amongst adults with hypertension.

## Introduction

Erectile dysfunction (ED) has been described as an important public health problem by the National Institutes of Health Consensus Development Conference Panel.^1^ It is defined as the inability to achieve or sustain an erection for satisfactory sexual activity.^2^ A variety of chronic illnesses such as cardiovascular diseases, diabetes mellitus, neurological diseases and depression are associated with higher rates of ED.^3^ In medical practice, physicians frequently attribute sexual problems to antihypertensive drugs and modify or discontinue medication regimens to address this concern.^4^ However, scientific evidence that links antihypertensive drugs to sexual dysfunction in a placebo-controlled trial is limited.^4^

The association of ED and vascular risk factors, including hypertension, raises the hypothesis that endothelial dysfunction is the common link.^5^ Hypertension is a traditional risk factor for cardiovascular disease.^6^ Recent analyses suggest that about 67% – 68% of men with hypertension have some degree of ED.^4^ In the United States, ED related to hypertension was found to be more severe in nature than ED in the general population.^5^ Apart from the negative effects of ED, more worrisome is the link between ED and hypertension. This is supported by Min et al., who found ED to be associated with markers of adverse cardiovascular prognosis and an independent predictor of severe coronary heart disease.^7^

A review of literature shows few studies on the relationship between ED and education level amongst hypertensive men. One such study was done by Abdulbari et al. in Qatari men.^8^ The study revealed that age, level of education, diabetes mellitus, occupation and duration of hypertension were significant predictors of ED. A similar study conducted in the Niger Delta region of Nigeria on the prevalence and risk factors for ED by Idung et al. observed that level of education, diabetes mellitus, hypertension and their medications played a major role as risk factors for ED.^9^ The authors reported that ED is one of the major social problems causing significant distress in men.^9^

It is with this background that this study was carried out to identify the relationship between level of education and ED amongst hypertensive men (aged 30–89 years) attending outpatient clinics (OPCs) at the Federal Medical Centre (FMC), Asaba, South-South Nigeria.

## Materials and methods

### Study design

A cross-sectional study design was carried out using systematic random sampling. Around 184 hypertensive men aged 30–89 years, attending the OPCs at FMC, Asaba, were recruited into the study from October 2015 to January 2016. Data were collected using a semistructured, interviewer-administered questionnaire adopted from the International Index of Sexual Health Inventory for Men (SHIM). Written informed consent was obtained from the consenting respondents. Confidentiality and privacy were ensured before data collection interview.

### Setting

The OPCs at FMC, Asaba comprise the children’s, adult and geriatrics clinics. The clinics are run by family physicians. This study was conducted in the adult and the geriatrics arms of the clinics.

### Study population

The study population were men between the ages of 30 and 89 years, attending the adult and the geriatrics clinics of the outpatient department of the FMC, Asaba. Adult hypertensive male patients 18 years and above, regardless of duration, smoking and alcohol consumption status, who consented to participate in the study were included, whilst those with a past history of surgical conditions that could cause ED were excluded from the study.

### Data collection

Data on sexual performance, educational and socio-economic status and other risk factors for ED were collected using a semistructured, interviewer-administered questionnaire adopted from the International Index of SHIM. With the SHIM scores obtained, ED was classified as follows: no ED = 22–25; mild ED = 17–21; mild to moderate ED = 12–6; moderate ED = 8–11 and severe ED = 1–7.

### Data analysis

Data cleansing was done, then entered into IBM Statistical Package for Social Sciences (SPSS) version 20 and analysed. Categorical data such as educational status and marital status were presented as percentages, whilst continuous variables such as age were expressed as means ± standard deviation. Bivariate analysis was carried out to test the association between ED and independent variables such as age, marital status and antihypertensive drugs. Logistic regression analysis was used to ascertain the relationship between predictor variables and ED.

### Ethical considerations

The ethical consideration was approved by the Research and Ethical Committee of the Federal Medical Centre Asaba, Delta State, Nigeria.

## Results

A total of 184 male hypertensive subjects were recruited in this study, and the sociodemographic characteristics were as shown in Table 1.

**TABLE 1:** The sociodemographic characteristics of the respondents.

Sociodemographic characteristics	Frequency (*n* = 184)	Percentage
**Age group (years)**		
30–39	17	93.0
40–49	51	27.7
50–59	46	25.0
60–69	41	22.3
70–79	26	14.1
80–89	3	1.6
**Level of education**		
None	11	6.0
Primary	32	17.4
Secondary	35	19.0
Post-secondary	44	23.9
University	62	33.7
**Marital status**		
Single	15	8.2
Married	138	75.0
Divorced	8	4.3
Cohabiting	7	3.8
Widowed	16	8.7

Note: Age group: mean age 55.1 (± 12.4 years). The total respondents with erectile dysfunction were 142 (i.e. 77.2% of the study population), as seen in Table 2.

**TABLE 2:** Prevalence of erectile dysfunction amongst respondents.

Erectile dysfunction	Frequency (*n* = 184)	Percentage	95% CI
Presence of ED	142	77.2	71% – 83%
Absence of ED	42	22.8	17% – 29%

**Total**	**184**	**100.0**	

ED, erectile dysfunction; CI, confidence interval.

The study showed that only 50 (27.2%) had severe ED as shown in Table 3.

**TABLE 3:** The degree of severity of erectile dysfunction amongst respondents.

Erectile dysfunction	Frequency (*n* = 184)	Percentage	95% CI
Severe ED	50	27.2	21.3% – 34.0%
Moderate ED	33	17.9	13.1% – 24.1%
Mild to moderate ED	37	20.1	15.0% – 26.5%
Mild	22	12.0	8.0% – 17.4%
Normal	42	22.8	17.4% – 29.4%

ED, erectile dysfunction; CI, confidence interval.

Across the different classes of antihypertensive drugs, only diuretics and calcium channel blockers had a statistically significant association with ED (*p* < 0.001 and *p* = 0.037, respectively), as seen in Table 4.

**TABLE 4:** Association between erectile dysfunction and different classes of antihypertensive drugs.

Erectile dysfunction	Different hypertensive medications					
	Yes		No		χ²	*p*
	*n*	%	*n*	%		
**ACE inhibitors**						
Severe	14	28.0	36	72.0	7.852	0.097
Moderate	10	30.3	23	69.7		
Mild to moderate	12	32.4	25	67.6		
Mild	4	18.2	18	81.8		
Normal	4	9.5	38	90.5		
**Calcium channel blockers**						
Severe	18	36.0	32	64.0	10.218	0.037*
Moderate	19	57.6	14	42.4		
Mild to moderate	20	54.1	17	31.8		
Mild	15	68.2	7	31.8		
Normal	28	66.6	14	33.3		
**Beta blockers**						
Severe	3	6.0	47	94.0	0.617	0.955
Moderate	2	6.1	31	93.9		
Mild to moderate	1	2.7	36	97.3		
Mild	1	4.5	21	95.5		
Normal	2	4.8	40	95.2		
**Diuretic**						
Severe	33	66.0	17	34.0	34.566	0.001*
Moderate	25	75.8	8	24.2		
Mild to moderate	24	64.9	13	35.1		
Mild	12	54.5	10	45.5		
Normal	7	16.7	35	83.3		
**ARBs**						
Severe	9	18.0	41	82.0	1.239	0.872
Moderate	7	21.2	26	78.8		
Mild to moderate	8	21.6	29	78.4		
Mild	3	13.6	19	86.4		
Normal	6	14.3	36	85.7		

ACE, angiotensin-converting enzyme.

* , Statistically significant.

The modifiable risk factors of alcohol consumption and cigarette smoking were not associated with ED, as a greater proportion of respondents who had ED did not consume alcohol (*p* = 0.912). Also, a greater proportion of respondents who suffered from different grades of severity of ED did not smoke cigarettes; however, this association was not statistically significant (*p* = 0.854). The grades of severity of ED increased with increasing body mass index (BMI); however, the association between ED and BMI was not statistically significant (*p* = 0.331). See Table 5 and 6.

**TABLE 5:** Association between erectile dysfunction and modifiable risk factors.

Erectile dysfunction	Modifiable risk factors (*n* = 184)					
	Yes		No		χ²	*p*
	*n*	%	*n*	%		
**Alcohol**						
Severe	20	40.0	30	60.0	0.991	0.912
Moderate	12	36.4	21	63.6		
Mild to moderate	12	32.4	25	68.6		
Mild	7	31.8	15	68.2		
Normal	29	69.0	13	31.0		
**Cigarette usage**						
Severe	20	40.0	30	60.0	1.377	0.854
Moderate	17	51.5	16	48.5		
Mild to moderate	15	40.5	22	59.5		
Mild	10	45.5	12	54.5		
Normal	17	40.5	25	59.5		

Note: For a year increase in the age of the respondents, the likelihood of having erectile dysfunction increases by the odds ratio of 0.888. This was statistically significant (*p* = 0.004, 95% confidence interval = 0.820 – 0.963).

**TABLE 6:** Association between erectile dysfunction and modifiable risk factors.

Weight	Body mass index							
	Underweight		Normal weight		Overweight		Obesity	
	*n*	%	*n*	%	*n*	%	*n*	%
Severe	1	2.0	12	24.0	16	32.0	21	42.0
Moderate	1	3.0	9	27.3	11	33.3	12	36.4
Mild to moderate	0	0.0	11	29.7	10	27.0	16	43.3
Mild	0	0.0	7	31.8	10	45.5	5	22.7
Normal	1	2.4	7	16.7	24	57.1	10	23.8

Note: χ² = 13.544, *p* = 0.331. For a year increase in the age of the respondents, the likelihood of having erectile dysfunction increases by the odds ratio of 0.888. This was statistically significant (*p* = 0.004, 95% confidence interval = 0.820–0.963)

Respondents with formal secondary education had ED more than those with no formal education. This was more likely to occur by the odds ratio of 15.943. This was statistically significant (*p* = 0.021, 95% confidence interval [CI] = 1.517–167.502).

The likelihood of having ED increased by the odds ratio of 0.105 amongst respondents who used diuretics compared to those who did not use. This was statistically significant (*p* = 0.001, 95% CI = 0.028–0.389). Having controlled for all other predictors, the antihypertensive calcium channel blockers with an odds ratio of 1.193 were not statistically significant (*p* = 0.772, 95% CI = 0.362–3.928) as seen in Table 7.

**TABLE 7:** Determinants of erectile dysfunction amongst the respondents.

Predictors	*p*	Odds ratio	95% CI for odds ratio	
			Lower	Upper
**Age of respondent (years)**	0.004	0.888	0.820	0.963
**Level of education**				
Nonformal	-	-	-	-
Secondary	0.021	15.943	1.517	167.502
Postsecondary	0.201	3.028	0.555	16.551
University	0.558	0.668	0.173	2.578
Marital status	0.079	0.215	0.054	1.175
**Duration of hypertension (years)**				
< 1	-	-	-	-
1–5	0.408	3.480	0.182	66.686
6–10	0.503	2.582	0.161	41.424
> 10	0.452	3.267	0.149	71.654
Diuretics	0.001	0.105	0.028	0.389
Calcium channel blockers	0.772	1.193	0.362	3.928
Stroke	0.998	0.000	0.000	0.000
Angina pectoris	0.559	2.246	0.110	45.686
Heart failure	0.096	0.112	0.008	1.476
Cataracts	0.889	0.806	0.040	16.367
Depression	0.106	0.145	0.014	1.507
Visual impairment	0.106	0.292	0.066	1.300
LUTS	0.997	0.000	0.000	-
Peripheral neuropathy	0.192	0.332	0.063	1.743
Constant	0.042	236.717	-	-

CI, confidence interval; LUTS, lower urinary tract symptoms

For a year increase in the age of the respondents, the likelihood of having ED increased by the odds ratio of 0.888. This was statistically significant (*p* = 0.004, 95% CI = 0.820–0.963).

Respondents with formal secondary education had ED more than those with no formal education. This was more likely to occur by the odds ratio of 15.943. This was statistically significant (*p* = 0.021, 95% CI = 1.517–167.502).

## Discussion

In this study, a greater proportion of the respondents were in the age group of 40–49 years; this may be explained by the fact that these age groups constituted the economically productive age group who might be able to afford the cost of health care. This finding of the economically productive age group being more ready to access health care was similar to the findings from a study carried out in 2002 by Valdivia in Peru to assess the magnitude and nature of socio-economic differences in the utilisation of outpatient health care services, which showed that utilisation amongst those who reported illness had a clear trend in favour of the wealthier.^10^ Also, another study in Mexico by Leyva-Flores et al. showed that economic barriers were the most frequent reasons for not using primary health care services.^11^

Three-quarters of the respondents were married; this was not surprising, as the mean age observed in the study was 55 ± 12 years. Most men would be expected to have been married at this age. This finding is similar to the finding in a study conducted by Idung et al. in the Niger Delta region of Nigeria on the prevalence and risk factors for ED. They found that ED increases with age and is more common amongst married and educated men.^9^ Over three-quarters, 147 (79.9%), of respondents were married in monogamous settings. This was probably so because the study was conducted at FMC, Asaba, which is in the South-South region of Nigeria where the major religion is Christianity.^12^ With most patients using the facility being Christians, it is expected that they are married to one wife in a monogamous setting.

Over one-third of the respondents were government employed. This is probably so because a greater proportion, 141 (76.6%), of the respondents had a secondary level of education and above, and the government of Nigeria is the highest employer of labour.^13^ This could possibly be the reason they could access health care on time; being economically productive and educated, they were able to feel free to discuss their problems with their doctors. Previous studies such as Willems et al., Cutler and Lieras-Muney have shown that patients from higher levels of education communicate more actively with their doctors.^14,15^ Willems et al. in 2005 concluded that communication is influenced in part by patients’ communication abilities and style, which depend largely on education.^14^ Similarly, Cutler and Lieras-Muney reported that an increase in cognitive ability resulting from education contributes significantly to the education gradient in health behaviours.^15^

The prevalence of ED amongst the respondents was found to be 77.2% (*n* = 142). The observed prevalence was higher than the rates of between 43.8% and 57.4% reported in previous studies in Nigeria.^16,17,18^ The increase in the prevalence rate discovered in this study could be explained by the fact that with increasing modernisation and awareness of the availability of treatment options for ED, more people are now bold enough to do away with stigmatisation and acknowledge their problem to their health care providers, and they are even more willing to participate in any research relating to ED, as was observed by the 100.0% response rate in this current study. The prevalence from this current study is similar to that observed in other studies such as Omisanjo et al., Burchaidt et al. and Giuliano et al.^19,20,21^ These findings were similar despite very different cultures, research instruments and methodologies. This clearly shows that ED is a worldwide problem.

In consideration of the association between age and degree of ED, this study showed that age was protective. This may be because of the fact that most of the participants were middle-aged. This was not supported by the result of the study carried out by Fatusi et al., which showed a significant relationship between age and ED.^17^ In a study by Shaeer et al. in 2003, they found that both severity and prevalence increased consistently with age, although their distribution differed across countries.^22^ Their study seemed to agree with the 2008 finding of Derogates et al. that the occurrence of sexual dysfunction is directly proportional to age in both sexes.^23^

In this study, the prevalence of ED was higher in patients with longer duration of hypertension, as well as with increasing years of being hypertensive. This was similar to other studies addressing this issue in hypertensive patients; they reported that ED is more frequent and severe in patients with long-standing hypertension (> 5–6 years) compared with recent onset of hypertension.^22,23,24,25,26^ Hence, appropriate counselling must be given to patients during the early stages of diagnosis of hypertension.

The present study showed that modifiable risk factors, which include history of cigarette smoking, drinking alcohol and BMI, were not significantly associated with ED. A greater proportion of respondents who had ED did not consume alcohol; also, a greater proportion of respondents who suffered from the different grades of ED did not smoke cigarettes. Although the grades of severity of ED increased with increasing BMI, the association was not statistically significant (*p* = 0.807). The finding in the current study differs from observations in previous studies, in which ED was found to be significantly associated with cigarette smoking and alcohol consumption.^27,28^ There are different opinions from studies on the association between obesity and ED. Whilst some studies reported no significant association,^29^ others demonstrated obesity as an independent risk factor for ED.^28,29,30,31,32,33,34,35^ Still others suggest that obesity in itself does not seem to be an underlying factor but imposes a risk to vasculogenic ED by developing chronic vascular disease.^36^

The finding in this current study showed that across the classes of antihypertensive medications, diuretics showed significant association, as a greater proportion of those on it had severe and moderate ED, which was statistically significant (*p* < 0.001; 95% CI = 0.028–0.389). Significantly increased association of ED with the use of diuretics could be because of the fact that those patients were on diuretics for a long time, as in our setting, diuretics are the first-line antihypertensive drugs. A similar study by Okeahialam and Obeka noted that hypertensive patients on thiazide tend to have more ED than the untreated, newly diagnosed group.^24^ There was also a significant association between ED and the calcium channel blockers in this study.

Regarding the association between ED and other comorbidities, the present study showed that comorbid medical conditions such as diabetes mellitus, history of stroke, angina pectoris, heart failure, peripheral neuropathy, past surgery and cataracts (which were highly significantly associated with ED on bivariate analysis) were not statistically significant on multivariate logistic regression as seen in Table 7. A greater proportion, 14 (28%), of the respondents with severe ED had stroke. There is a direct relationship between the severity of ED and incidents of stroke. These findings were similar to the findings from studies done by Khatib et al., who found that patients with history of stroke and presence of peripheral neuropathy were associated with ED.^37^ Abdulbari et al. also found an association between history of stroke and other associated comorbidities with ED.^38^ The reason could be because ED is a vascular problem.^39^

A greater proportion, 26 (52.0%), of respondents with severe ED had heart failure compared to the other grades of severity of ED; therefore, with increasing grades of ED, there was increase in heart failure. This was similar to the recent studies done by Baraghoush et al. and Alberti et al., in which they found that there was a link between ED and heart failure.^40,41^ It could be because they shared similar risk factors and common pathogenic traits. As a result, proper medical check-up and follow-up should be done for the elderly hypertensive males with risk of having heart failure, so as to reduce complications and improve their quality of life.

Over half, 28 (56.0%), of the respondents with severe ED had lower urinary tract symptoms (LUTS) compared with the other proportions of respondents with LUTS in the other grades of ED. This was similar to several studies that highlighted the association between ED and other medical conditions such as LUTS.^42,43^

Medical conditions such as myocardial infarction, depression and past surgery were not statistically significant. This may be due to the fact that the number of respondents with these medical conditions who participated in the study was low. This finding was not concordant with the findings from other studies.^29,44,45^

## Limitations

Erectile dysfunction is regarded as a couple’s disorder. However, the interview in this study focused only on the male partner. Therefore, the female partners’ perceptions and the degree of affectation of the family dynamics cannot be fully ascertained in this study.

## Conclusion

The present study showed that formal education up to the secondary level and use of diuretics are significant predictors of ED amongst hypertensive men attending the OPC at FMC.
